# A Lateral Differential Resonant Pressure Microsensor Based on SOI-Glass Wafer-Level Vacuum Packaging

**DOI:** 10.3390/s150924257

**Published:** 2015-09-21

**Authors:** Bo Xie, Yonghao Xing, Yanshuang Wang, Jian Chen, Deyong Chen, Junbo Wang

**Affiliations:** 1 State Key Laboratory of Transducer Technology, Institute of Electronics, Chinese Academy of Sciences, Beijing 100190, China; E-Mails: xiebo11@mails.ucas.ac.cn (B.X.); xingyonghao13@mails.ucas.ac.cn (Y.X.); wangeomshuang13@mails.ucas.ac.cn (Y.W.); chenjian@mail.ie.ac.cn (J.C.); 2 University of Chinese Academy of Sciences, Beijing 100190, China

**Keywords:** resonant pressure sensor, differential frequency output, vacuum packaging, anodic bonding, wire interconnection, mask-free metallization

## Abstract

This paper presents the fabrication and characterization of a resonant pressure microsensor based on SOI-glass wafer-level vacuum packaging. The SOI-based pressure microsensor consists of a pressure-sensitive diaphragm at the handle layer and two lateral resonators (electrostatic excitation and capacitive detection) on the device layer as a differential setup. The resonators were vacuum packaged with a glass cap using anodic bonding and the wire interconnection was realized using a mask-free electrochemical etching approach by selectively patterning an Au film on highly topographic surfaces. The fabricated resonant pressure microsensor with dual resonators was characterized in a systematic manner, producing a quality factor higher than 10,000 (~6 months), a sensitivity of about 166 Hz/kPa and a reduced nonlinear error of 0.033% F.S. Based on the differential output, the sensitivity was increased to two times and the temperature-caused frequency drift was decreased to 25%.

## 1. Introduction

Silicon resonant pressure microsensors have been widely used in the automotive industry, medical instrument, aerospace and military fields due to their high accuracy, good long-term stability and quasi-digital output [1,2,3]. However, conventional resonant pressure sensors have to find a balance between sensitivity and nonlinearity error (e.g., high deflection of the pressure-sensitive membrane leads to large nonlinear error). Previously, a SOI-based electromagnet resonant pressure microsensor [4,5] with dual resonators as a differential structure was proposed to improve the linearity and sensitivity. The two resonators with reversed pressure reaction were vacuum packaged with a glass cap using anodic bonding such that the common drift were also reduced using the differential frequency output of the two resonators.

However, these devices are based on electromagnetic excitation and detection, requiring permanent magnets, which is not desirable in specific applications (e.g., airplanes) due to the concerns of potential electromagnetic interferences. Besides, wire interconnections with through-glass-via-holes (TGVs) fabricated by sandblasting or ultrasonic machining cannot define via holes with high spatial resolutions, and are not compatible with microfabrication processes [6,7]. Furthermore, cracks were formed at the edges of via holes which further reduced the reliability of vacuum packaging.

Although resonators driven by electrostatic forces are featured with low thermal noises, low power consumption and no concern of magnetic interferences, the gap between capacitive plates was reduced to several micrometers to improve the signal to noise ratio (SNR). Thus, the issue of pull-in damages should be further addressed during the process of anodic bonding. Specifically, pull-in damages are caused by the high voltage applied to the SOI wafer during the anodic bonding and corresponding voltage difference between electrode plates. For the case of vertical pull-in damages [8], the distance between the glass cap and silicon microstructures can be enlarged to address this issue. However, this method is not suitable for resonators working in the lateral mode, since narrow gaps between capacitive plates are usually preferred for the concern of driving force.

To solve these problems, three improvements were implemented in this paper: (1) via holes on the handle layer instead of TGVs were fabricated for wire interconnections, which can lead to high vacuum anodic bonding with long-term stability; (2) the device layer was deposited by a metal film forming an equal potential through those via holes, which can effectively address the issue of lateral pull-in damages of movable parts during anodic bonding; (3) a mask-free method of selectively gold patterning on the highly topographic surface with via holes was developed to form gold pads in via holes for wire bonding.

## 2. Sensor Design

**Figure 1:**
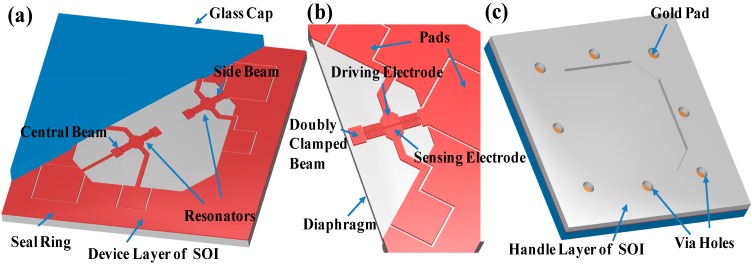
(**a**) Top view of the proposed pressure micro sensor, including a patterned SOI wafer and a glass cap, which are bonded together as vacuum packaging; (**b**) Detailed view of the resonator, which consists of a doubly clamped resonant beam, a driving electrode, and a sensing electrode. The resonator works in the lateral mode which is excited based on electrostatic forces and detected based on capacitance; (**c**) Bottom view of the micro sensor where gold pads on the device layer are metalized for interconnection through via holes formed on the handle layer.

The conceptual diagram of the proposed resonant pressure microsensor is shown in Figure 1. The sensor chip with a size of 10 × 10 × 1 mm^3^ consists of a diaphragm (5.2 mm × 5.2 mm) for pressure sensing, two resonators, a sealing ring, and a glass cap. Each resonator is made up of a doubly clamped resonant beam (length: 1200 μm, width: 20 μm, and thickness: 40 μm) and two parallel plates positioned on both sides of each beam (with a gap distance of 2 μm) as driving and sensing electrodes. The two beams are clamped on the diaphragm along the diagonal direction, which are termed as the “central beam” (the beam located at the center of the diaphragm) and the “side beam” (the beam around the diaphragm boundaries), respectively. The two parallel plates, functioning as the driving electrode and the sensing electrode, are responsible for the excitation and detection of the resonant beam in a lateral mode. The resonators are vacuum packaged in a micro chamber by a glass cap with a sealing ring around the bonding edge. The vacuum micro chamber not only provides a reference pressure, but also serves as a low damping environment for the resonators. Furthermore, wire interconnections are realized by via holes of 500 μm in diameter on the handle layer of the SOI wafer, which are directly linked to the pads of each electrode on the device layer.

The two resonators work in a differential mode. When the pressure is applied to the diaphragm, the deflection of the diaphragm builds up compression stresses on the side beam and tensile stresses on the central beam, leading to the resonant frequency shifts of the central beam (ƒ_1_, resonant frequency increases) and the side beam (ƒ_2_, resonant frequency decreases). The differential frequency output (ƒ_1_ − ƒ_2_) of the two resonant frequencies is then translated into the pressure under measurement. Note that the differential setup can address the issue of environmental temperature disturbances since temperature variations can lead to similar resonant frequency shifts. Besides, the sensitivity could also be amplified because of the reversed frequency translated directions of the two resonators.

## 3. Device Fabrication

**Figure 2:**
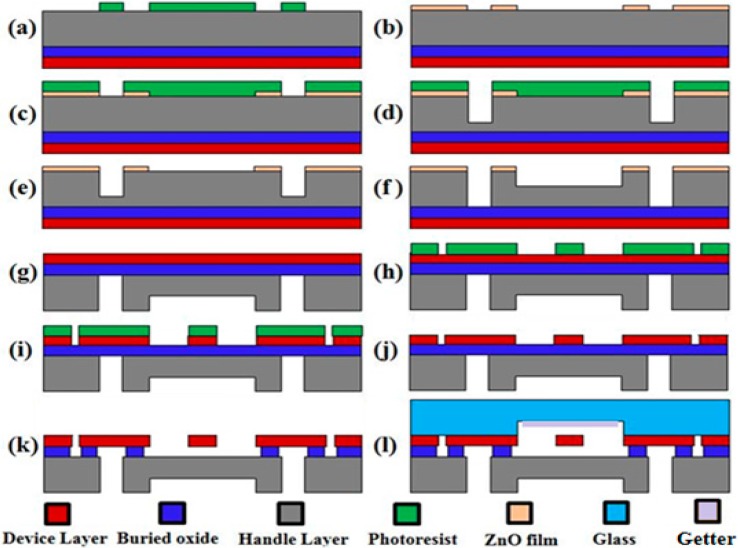
Device fabrication flow charts: (**a**,**b**) Patterning the ZnO film based on e-beam evaporation and lift-off process; (**c**,**d**) Patterning photoresist on the ZnO film as a mask to form via holes; (**e**,**f**) Forming via holes and diaphragm using the patterned ZnO film as hard mask; (**g**–**j**) Fabricating resonators on the device layer based on photolithography and dry etching; (**k**) Removing the oxide layer in vapor HF to release resonators; (**l**) Vacuum packaging of the fabricated silicon wafer and the glass cap wafer using anodic bonding.

The device fabrication was started from a 4-inch SOI wafer with a 40 μm P-type < 100 > device layer, a 2 μm SiO_2_ sacrificed layer, and a 300 μm N-type handle layer. The silicon layers were heavily doped with extremely low resistance of 0.0005 to 0.001 Ohm·cm for electrical connection. Figure 2a–f shows the fabrication processes of diaphragms and via holes using photoresist and ZnO film as multiple masks. AZ5214E photoresist was firstly spun and patterned on the handle layer of the SOI wafer. Then, a ZnO film of 100 nm was deposited by e-beam evaporation (AXXIS, Kurt J. Lesker, Pittsburgh, PA, USA) and patterned by the lift-off process to form the diaphragms and via holes [9,10], as shown in Figure 2a,b. As the next step, AZ4620 photoresist of 4 μm was spun and patterned on top of the ZnO film layer, which functioned as the etching mask to form via holes in the handle layer. The via holes were firstly dry etched to a depth of 200 μm using the BOSCH processes (SF6: 700 sccm, 7 s; C4F8: 140 sccm, 2 s; Power: 1800 W) [11,12]. Then, the AZ4620 photoresist was removed, and the patterned ZnO layer was used as the second mask to form the pressure-sensitive diaphragms of 120 μm, with the remained silicon in the via holes etched to the stop layer (see Figure 2e,f).

As to the fabrication of resonators on the device layer, the processes were much straightforward. The remaining ZnO film in Figure 2f was firstly removed in HCl solution and the wafer was cleaned in piranha solution for 30 min at 120 °C. After AZ4620 photoresist was spun on the device layer with a speed of 4000 rpm, soft-baked at 95 °C for 3 min on a hotplate, the photoresist was exposed to UV light (12 s, 15 mW/cm^2^), developed in AZ300MF for 150 s, and hard-baked on a hotplate (120 °C, 10 min). The device layer was also dry etched to the buried oxide layer with BOSCH processes again to form the resonators in Figure 2g–i. In order to ensure the verticality and smoothness of the sidewalls of the gap, a lower etching rate was used (SF6: 350 sccm, 5 s; C4F8: 250 sccm, 2 s; Power: 1500 W). Furthermore, due to the Lag effects [13,14] that the etching rate in the narrow gaps was much lower than that in the parts with bigger exposed areas, a prolonged etching period of 20 min was used to ensure the formation of 2 μm capacitor gaps. Then, the resonators were released in HF vapor where the oxide layer was removed (see Figure 2k), before that the photoresist on the silicon was removed and the wafer was cleaned. Finally, vacuum packaging was realized by bonding the patterned SOI wafer to a glass cap wafer with a cavity depth of 120 μm using a bonding voltage of 1000 V in an anodic bonding machine (SB6e, SUSS, Munich, Germany). Note that the glass cap wafer was deposited with a gas getter film [15] before anodic bonding to get rid of the gases produced in the bonding process to maintain the high vacuum environment in the micro chamber. 

**Figure 3:**
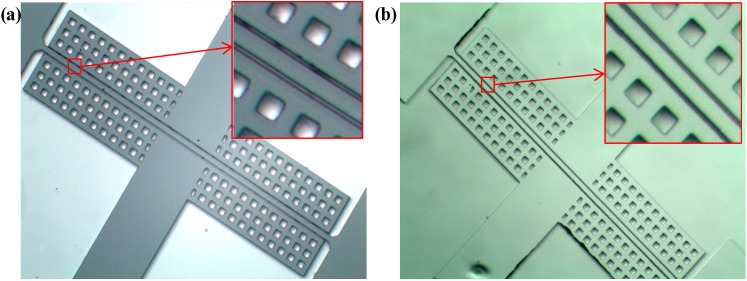
(**a**) Lateral pull-in damage (collapses between the resonant beam and driving/sensing electrode) after the step of anodic bonding; (**b**) The deposition of an Al film on the handle layer before anodic bonding can effectively avoid the pull-in damage.

As to the results of device fabrication, lateral pull-in damages (collapse between the driving/sensing electrodes) were found after the application of 1000 V voltage during the step of anodic bonding, as shown in Figure 3a. The possible reason is the existence of a voltage difference between driving electrode and sensing electrode during SOI-glass anodic bonding [16]. Then an Al film of 200 nm was deposited on the device layer through those via holes on the handle layer to eliminate the potential difference of the resonators, which effectively addressed the issue of lateral pull-in damage, as shown in Figure 3b.

## 4. Metallization and Wire Interconnection

**Figure 4:**
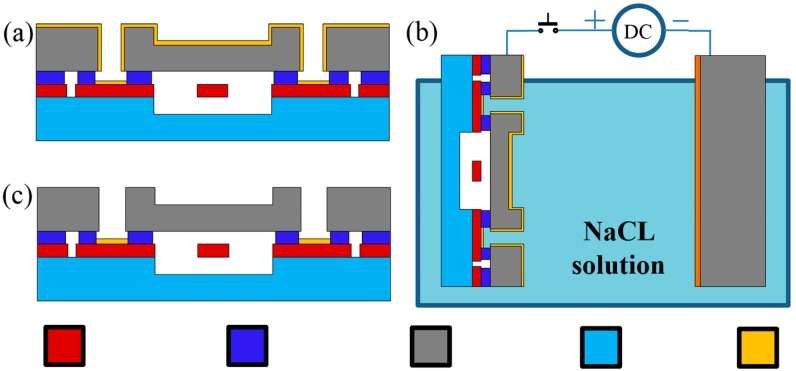
The schematic of selective pad patterning in via holes based on the electrochemical dissolution of gold. (**a**) Cr/Au film deposition; (**b**) Electrochemical etching. (**c**) Gold portions in via holes were preserved.

In this study, a new process of metallization and wire interconnection was developed based on an electrochemical etching approach [17,18] to pattern the Au film on highly topographic surfaces without masks (see Figure 4). More specifically, a Cr/Au film of 30 nm/200 nm was sputtered on the handle layer as well as the exposed device layer of the patterned SOI wafer (see Figure 4a). Then the Cr/Au film on the handle layer was connected to the anode of a DC source and a second silicon substrate deposited with Pt was connected to the cathode. Both electrodes were immersed in a NaCl solution, enabling the selective removal of Au in the chloride (Cl^−^) solution at anode (see Figure 4b). Since the Au film on the device layer was not connected with the anode, the gold film on the device layer was left intact, which can be further used for wire interconnections (see Figure 4c).

During this step, there is a concern of potential electrical connections between the handle layer and the device layer in the sputtering process. In order to deal with this issue and break the electrical connections between the device layer and the handle layer, two optimization steps were conducted: (1) the etching uniformity was improved by removing the bubbles in the etchant solution based on continuously stirring during the etching process, which can remove the gold connections between the device layer and the handle layer since its thickness of gold at this portion is much thinner than the gold film deposited on the device and the handle layer; (2) the thin connecting Cr layer beneath the gold layer was also removed by adding HCl into the NaCl solution at 60 °C.

**Figure 5:**
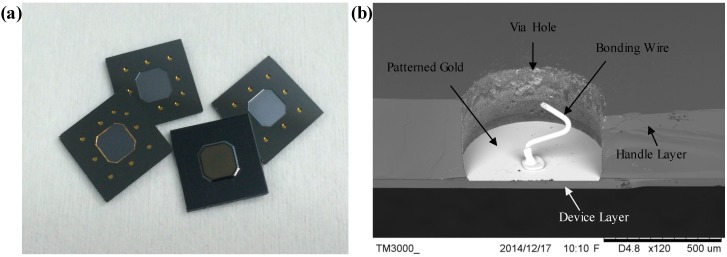
(**a**) A picture of the micro-fabricated devices; (**b**) A SEM picture of a via hole after wire bonding, where the gold portion on the handle layer was thoroughly removed with the gold portion left on the device layer for wire bonding.

The fabricated device is shown in Figure 5a and a SEM picture of a via hole after wire bonding is shown in Figure 5b. Experimental results confirmed that the gold portion on the handle layer was thoroughly removed with the gold portion only left on the device layer for wire bonding. It is confirmed that the proposed method enables the selective metallization on the device layer and the successful formation of electrical interconnections with the surrounding world.

## 5. Sensor Performance Characterization

**Figure 6:**
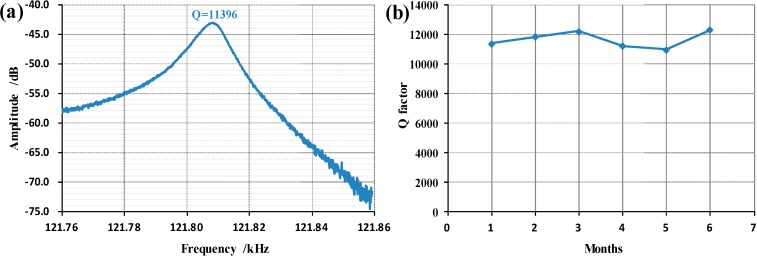
(**a**) The quality factor of the proposed pressure micro sensor was quantified as 11,396; (**b**) The quality factor was higher than 10,000 within six months of device fabrication, which confirms the reliability of the SOI-glass wafer-level vacuum packaging.

The fabricated pressure micro sensor was characterized using an E5061B Network Analyzer (Agilent, Palo Alto, CA, USA) where the quality factor was obtained as an indicator of the status of the vacuum packaging. Figure 6 reports the obtained quality factor of 11,396, which was shown to be higher than 10,000 within six months of continuous device functioning, confirming the reliability of the SOI-glass wafer-level vacuum packaging.

**Figure 7:**
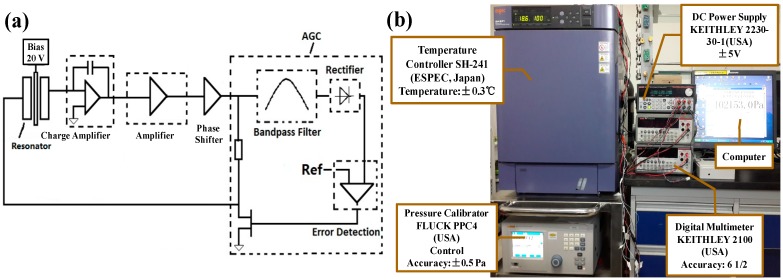
(**a**) The schematic of the closed-loop circuit used as a self-oscillator for frequency detection; (**b**) The setup used to characterize the sensor performance as functions of both pressure and temperature.

In order to detect the resonant frequency portably, a closed-loop circuit producing the self-oscillation signal was developed, as shown in Figure 7a. The microbeam was biased with a voltage of 20 V to charge the capacitor formed by the resonant beam and the sensing electrode. The beam vibration changed the value of the capacitor, leading to the charge variations on the sensing electrode. Then, the charge amplifier transferred the charges on the sensing electrode into a voltage signal as the output. The voltage was then depressed and sent to the driving electrode for the actuation of the resonant beam in a closed-loop manner. In order to maintain the stable vibration of the resonant beam, an automatic gain controlled circuit (AGC), consisting of a band pass filter, a rectifier, an error detector, and a voltage controlled resistor, was integrated into the loop.

The setup used to calibrate and characterize the micro pressure sensor is shown in Figure 7b. The microsensor with the closed-loop circuit was sealed in a chamber, leaving a valve connected to a pressure calibrator (PPC4, Fluke, Everett, WA, USA) to provide the required pressure. Then, the chamber was put into a temperature controller (SH241, ESPSC, Osaka, Japan) to quantify the device performance as a function of environmental temperatures. Two digital multimeters (2100, Keithley, Cleveland, OH, USA) were used to record the frequencies of the closed-loop circuit. The setup was used to calibrate the sensor in a temperature range from −40 °C to 60 °C and a pressure range from 50 kPa to 110 kPa with intervals of 20 °C and 10 kPa, respectively. Besides, the temperature drift was also quantified when the microsensor was controlled at a constant pressure.

**Figure 8:**
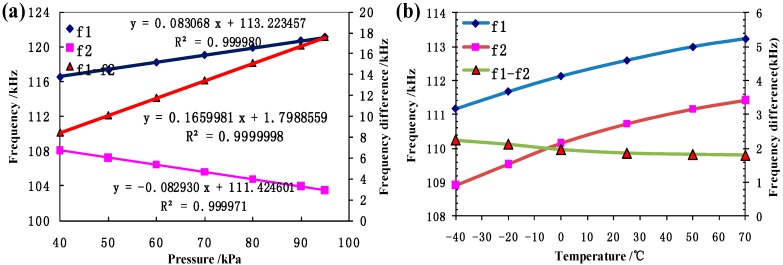
(**a**) The intrinsic resonant frequencies of these two resonators as a function of pressure, producing a sensitivity of about 166 Hz/kPa and a linearity with a coefficient of 0.9999998 based on the differential setup; (**b**) Two resonators demonstrated similar temperature drifts and the temperature caused frequency drift was reduced by the differential output.

**Figure 9:**
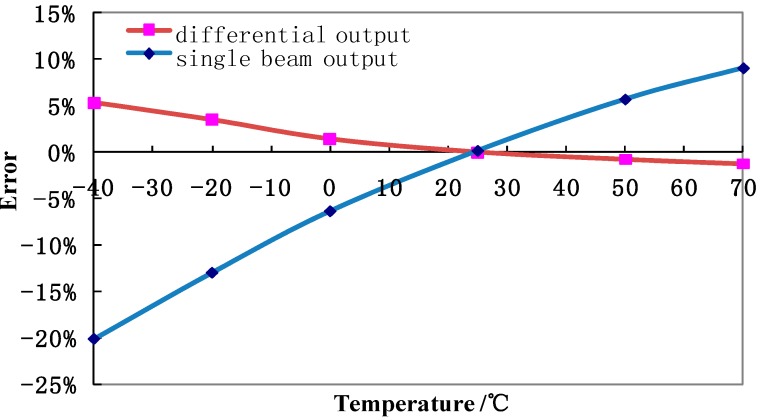
In response to temperature changes, the quantified errors using the differential frequency output were about four times smaller than the values obtained using the single resonator.

Figure 8a shows the intrinsic resonant frequencies of the two resonators as a function of pressure under measurement, producing a two-fold sensitivity of about 166 Hz/kPa and a nonlinear error of 0.033% F.S. based on the differential frequency output. Figure 8b shows that two resonators demonstrated comparable temperature drifts under constant pressure supply and the temperature caused frequency drift was also reduced with the differential output. Figure 9 shows the temperature response of the proposed pressure microsensor, in which the temperature based error was reduced from 20% to 5% using the differential frequency as output.

**Figure 10:**
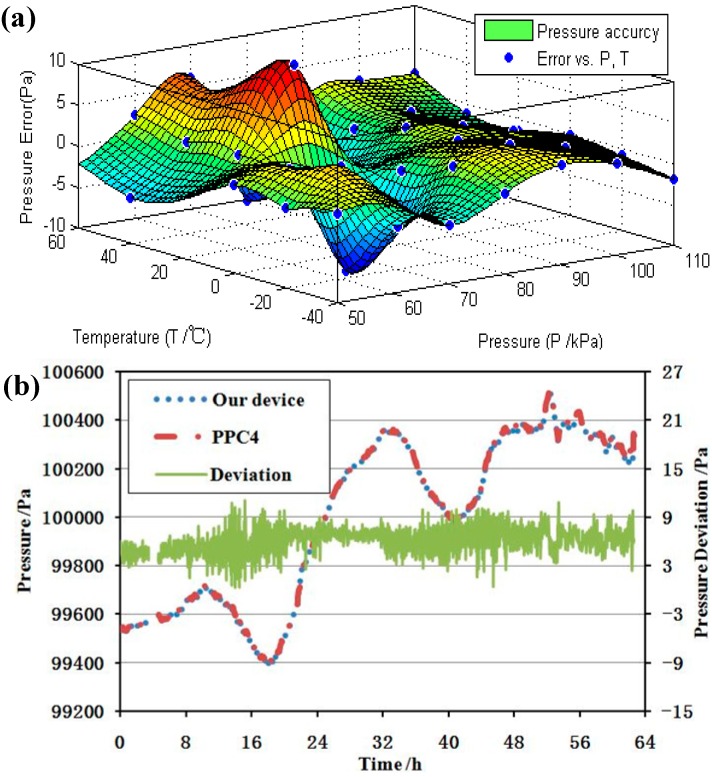

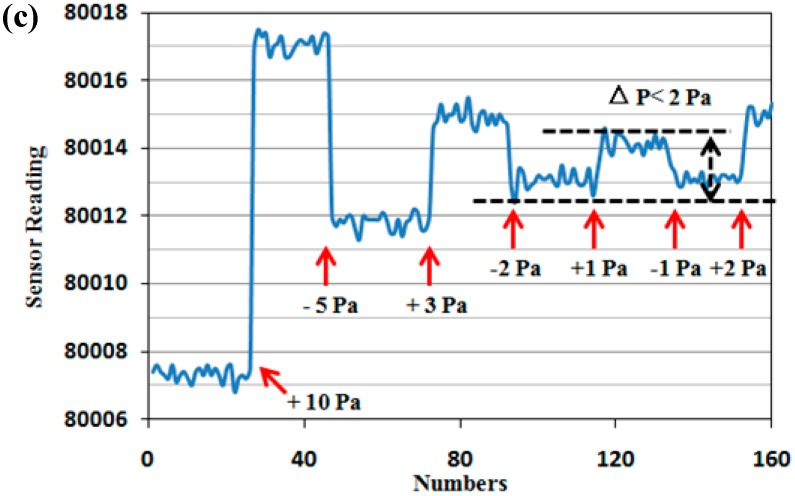
(**a**) The quantified error of the developed micro sensor in a temperature range from −40 °C to 60 °C and a pressure range from 50 kPa to 110 kPa; (**b**) The compared results of the proposed micro pressure sensor in the study and a quartz resonant pressure sensor in Fluck Fluke PPC4 in response to environmental pressure variations; (**c**) The performance of the developed pressure microsensor in response to regulated small pressure changes (+10 Pa, −5 Pa, +3 Pa, −2 Pa, +1 Pa, −1 Pa, +2 Pa).

The differential setup helped to improve the pressure sensitivity and reduced the temperature drift, simplifying the step of temperature compensation. After compensation, the detection error of the microsensor was about 10 Pa, as shown in Figure 10a. A comparison of two-day pressure tracing results of the proposed microsensor and a commercially available high-accuracy pressure controller with an imbedded quartz resonant pressure sensor was illustrated in Figure 10b.

The data indicates that they the two micro sensors had identical pressure shifts and a constant deviation as a function of time, confirming the accuracy of the developed microsensor within 10 Pa. The resolution of the sensor was also tested by connecting the proposed microsensor to the PPC4 controller, which provided a reference pressure with a fluctuation of ±0.5 Pa. The pressure was controlled at 80 kPa with a series of changes of +10 Pa, −5 Pa, +3 Pa, −2 Pa, +1 Pa, −1 Pa, and +2 Pa. The results in Figure 10c show that the resolution of the developed pressure microsensor was higher than 2 Pa. As shown in Table 1, the proposed sensor demonstrated an equivalent performance with previously reported counterparts. Note that the quantified Q factor was lower than previously reported devices, which may result from squeezing film damping caused by the narrow capacitor gap.

**Table 1:** A comparison of device performance based on literature.

Reference	Q Factor	Pressure Range	Linearity	Sensitivity	Accuracy
C.J. Welham [1]	50,000	0~200 kPa	-	112.6 Hz/kPa	-
K. Harada [19]	50,000	0~100 kPa	-	~360 Hz/kPa	0.01% F.S.
R. Sen [20]	1772	100~400 kPa	0.045% F.S.	20.77 Hz/kPa	0.05% F.S.
C.-F. Chiang [21]	-	30~100 kPa	-	33.85 Hz/kPa	0.2% F.S.
Z. Luo [4]	22,000	50~100 kPa	0.02% F.S.	89.86 Hz/kPa	0.02% F.S.
Our device	11,000	50~110 kPa	0.033% F.S.	166 Hz/kPa	0.02% F.S.

## 6. Conclusions

This paper presented the fabrication and characterization of a lateral resonant pressure microsensor based on wafer-level vacuum packaging. A double-masks approach based on the patterned ZnO film and photoresist was used to etch via holes and pressure-sensitive diaphragms with different depths. The deposition of a metal layer on the device layer through the via holes before anodic bonding was used to effectively address the issue of lateral pull-in damages. The mask-free gold etching method based on the electrochemical principle was confirmed to selectively pattern the gold pads in via holes for wire bonding. Using the proposed dual resonators as a differential setup, the pressure sensor showed high linearity with a coefficient of 0.9999998 in the pressure range from 50 kPa to 110 kPa and the nonlinear error was reduced from 0.22% F.S. to 0.033% F.S. Due to the differential output, the microsensor doubled the pressure sensitivity and reduced by one-quarter of the temperature based influence, which makes the microsensor can be temperature-compensated without difficulties, producing an accuracy of 10 Pa and a resolution of 2 Pa in the pressure range from 50 kPa to 110 kPa.

Although the sensor chip was successfully designed, fabricated, and characterized with high accuracy, the sensor is still bulky to some extent. Further work will focus on the optimization of packaging, including the dimension reduction in via holes, pads, and sealing rings to improve the batch in one wafer. Besides, the long-term stability of the proposed pressure microsensor will be characterized where the issue of assembly stresses will be carefully addressed.
